# Supplementary material for: Contemporaneous symptom networks for multidimensional symptom experience in lung cancer survivors of immunotherapy: A network analysis
Source: PLoS One. 2025 Jul 10;20(7):e0327804. doi: 10.1371/journal.pone.0327804 (PMC12244778; doi:10.1371/journal.pone.0327804)
Supplement: Attachment — Submitted filename: Response to Reviewers.docx [file pone.0327804.s005.docx]

Dear Editor,

Thank you for your kind letter and for the reviewer’s comments concerning our manuscript entitled ‘Contemporaneous symptom networks for multidimensional symptom experience in lung cancer survivors of immunotherapy: a network analysis’ (ID: PONE-D-24-51139).

The reviewer’s comments were highly insightful and enabled us to greatly improve the quality of our article. We have studied the comments carefully and have made corrections, which we hope meet with approval. Revisions and point-to-point responses to each of the comments of the reviewers are as follows:

Comments to the author:

**Reviewer #1**

**Reviewer #1: Very important topic and well written overall. please address the below suggestions
Comment 1:Introduction: Add one paragraph regarding what are known in symptoms side effects in lung cancer, what are existing literature in ymptom cluster in lung cancer, what are existing literature or none regarding network analysis in symptoms in lung cancer.**

**Reply:** Thank you very much for recognizing our choice of topic. Thank you for your valuable feedback on the Introduction. We fully acknowledge your suggestions and have incorporated content related to symptom side effects in lung cancer, symptom cluster in lung cancer, and network analysis of symptoms in lung cancer into the revised Introduction section. These additions aim to provide a more comprehensive foundation for our research background.

**Comment 1.1: Symptoms side effects in lung cancer**

**Reply**: Please refer to lines 64–77 in the revised manuscript for information on the side effects of lung cancer symptoms:

Lung cancer patients generally suffer from a multidimensional symptom burden, with fatigue (72-89%), dyspnea (58-76%), pain (52-68%), and psychological distress as the main symptoms, significantly affecting treatment adherence and quality of life [4-6]. Longitudinal studies have shown that symptom peaks in postoperative adjuvant therapy patients are synchronized with the chemotherapy cycle, with cough (83% incidence), dry mouth (76%), and sleep disturbances (68%) being most prominent before surgery [7]. During radiation therapy, fatigue, shortness of breath, and coughing up sputum are the main challenges [8]. The severity of symptoms in lung cancer patients is moderated by a variety of factors such as age, gender, and economic income. Studies have shown that the prevalence of digestive and mood symptom clusters was 32% higher in female patients compared to males, the risk of mood psychological symptoms was increased by 45% in the low-income group, and the VAS scores of fatigue and sleep disturbances in the older patients (>65 years of age) were 1.8 points higher than those in the younger group [9].

**Comment 1.2: Sympotm cluster in lung cancer**

**Reply:** Literature related to symptom clusters in lung cancer patients has been increased, please refer to lines 106-136 in the revised manuscript:

Prior research has indicated a stage-specific agreement for the investigation of the dynamic evolutionary pattern of symptom clusters associated with lung cancer treatment and their clinical management approaches. The pain-fatigue-sleep disorders-distress tetralogy (68-82%) was the most prevalent symptom cluster in the perioperative stage, according to treatment stage-specific analysis, and its pathophysiological basis might be linked to a systemic inflammatory response brought on by surgical trauma[19, 20];The dyspnea-fatigue-anxiety triangle predominates during radiotherapy, and V/Q dysregulation due to radiological lung injury forms a bidirectional modulation with vagal-mediated anxiety[21];The chemotherapy phase differentiated a three-dimensional symptom cluster of lung cancer-specific (hemoptysis/chest pain), psychological (depression/cognitive deficits), and nutritional (cachexia/abnormal taste) symptoms, in which platinum-based chemotherapeutic regimens induced activation of TRPV1 channels leading to the neuropathic pain characteristic of the symptom cluster[22];During the synchronized treatment period, a cluster of radiation pneumonitis symptoms coexist with a pain-pharyngeal pain dichotomy, which is caused by cytokine storm and disruption of the mucosal barrier[23]. Longitudinal investigations have also demonstrated the dynamic evolution of symptom clusters in individuals with lung cancer, with significant remission occurring at 6 weeks and peak symptom severity occurring 3 weeks following radiation therapy[24]. A prospective cohort study by the Gift team (N=358) confirmed that multidimensional symptom clustering resulted in a 42% reduction in severity scores at 6 months of follow-up, suggesting that the acute phase (8-12 weeks after treatment initiation) is the optimal window for intervention[25]. However, existing studies have dual limitations: Traditional symptom clustering analysis overly relies on statistical co-occurrence and ignores non-linear interactions between symptoms compared to isolated symptoms; the unique symptom network triggered by immune checkpoint inhibitor therapy has not been systematically resolved, and its characteristics may show phase-variable patterns depending on the degree of PD-1/CTLA-4 inhibition. Establishing a new paradigm for symptom cluster research based on network pharmacology and dynamic system modeling will be a key path to breaking through the current management bottleneck.

**Comment 1.3: Network analysis of symptoms in lung cancer**

**Reply:** Literature related to network analysis of symptoms in lung cancer has been increased, please refer to lines 154-178 in the revised manuscript:

Network analysis, as an important methodological tool for complex systems research, has been extended from the traditional field of psychopathology [30] to the study of chronic disease symptomatology in recent years, and has shown unique parsing value especially in the fields of HIV/AIDS [31] and malignant tumors [32] domains showing unique parsing value. The method can systematically reveal the dynamic interplay mechanism of multidimensional symptom clusters and their clinical evolution by constructing symptom interaction networks [33]. Currently, there are few studies on network analysis in the field of lung cancer, and the existing research mainly focuses on the symptoms of patients during the perioperative period and chemotherapy. Luo et al.[34] investigated 1,255 lung cancer patients undergoing chemotherapy and identified four symptom clusters: fatigue, gastrointestinal, psychoneurological, and respiratory. Their central symptoms were fatigue, vomiting, distress, and hemoptysis, respectively, with bridge symptoms identified as pain, vomiting, dry mouth, and shortness of breath. Wang et al.[35] conducted a survey using the Chinese version of the M.D. Anderson Symptom Inventory (MDASI) among 301 perioperative lung cancer patients. They found that the most prevalent symptoms during the perioperative period were pain, fatigue, and shortness of breath, with shortness of breath identified as the core symptom. The study recommended that healthcare providers prioritize interventions targeting this core symptom while also focusing on pain and fatigue to enhance the efficiency and precision of symptom management in perioperative lung cancer care. In the future, it will be essential to integrate machine learning with multimodal data (e.g., biomarkers, radiomics) to develop real-time monitoring tools, validate precision management strategies through dynamic network-driven intervention trials, and promote innovation in lung cancer symptom management via interdisciplinary collaboration.

**Comment 2: General information questionnair -> this is typo, correct please**

**Reply:** Thank you for your careful review of our manuscript and for pointing out the spelling errors in the ' General information questionnair '. We are very sorry for such basic errors. We have corrected it to ' General information questionnaire ' in the revised version according to your suggestion (Please see line 236 of the revised manuscript.) and checked the full text to ensure the accuracy of the use of terms. We sincerely thank you for your valuable comments on improving the quality of this manuscript. If there is any other need for further clarification or modification, please feel free to inform.
**Comment 3:methods: CLARIFY when the symptoms are assessed, after treatments? or durng? or pre treatments?clarify authors used the SEVERITY of symptoms, or prevalence of symptoms for this network analysis As this is HUGE different analysis .**

**Comment 3.1: CLARIFY when the symptoms are assessed, after treatments? or durng? or pre treatments?**

**Reply:** Thank you very much for this question. We have supplemented the symptom assessment time.(Please refer to lines 257-263 in the revised manuscript.):

This study used a cross-sectional design, with data collected within 30 ± 7 days after patients completed immune checkpoint inhibitor (ICI) therapy. This time window was chosen for two reasons: first, the timing of immune-related adverse effects is unpredictable (median ranging from 1 to 6 months)[39], and irAEs may continue to develop beyond the end of treatment; and second, this period captures the peak symptom load at the beginning of the end of treatment[40].

**Comment 3.2: clarify authors used the SEVERITY of symptoms, or prevalence of symptoms for this network analysis As this is HUGE different analysis**

**Reply:** Thank you for your valuable comments. It is important to clarify whether symptom severity or symptom prevalence was used in the network analysis for the interpretation of the network analysis. The network analysis in this study used severity of symptoms for the reasons described in lines 332-353 of the revised manuscript.

The value of network analysis as an important methodological tool for complex systems research is not only limited to describing single-dimensional features such as the incidence or severity of symptoms, but also in revealing the dynamic interaction mechanisms among symptoms through topological indicators (e.g., centrality, intensity, tightness, and mediativity)[45]. This advantage is particularly prominent in the study of immunotherapy-related symptoms: as immune-related adverse events (irAEs) often present a subclinical persistence, the traditional dichotomous variable (presence/absence) loses the information about the gradient of symptom intensity, whereas continuous severity scores capture the dynamic evolution of the symptom load with greater precision. Moreover, from the methodological point of view: 1) the correlation between severity score and patients' quality of life was significantly higher than that of the incidence index, suggesting that it has more prognostic predictive value[46]; 2) the biased correlation network, also known as Gaussian graph model, constructed by network analysis requires the input of continuous variables, while the incidence data need to use Logistic network model[28]; And the severity gradient can reflect both the intensity dimension and the time dimension (e.g., fluctuation frequency, duration) of the symptoms, providing a richer basis for decision-making for dynamic intervention. Therefore, the symptom network constructed in this study reflects the covariate pattern of symptom severity rather than the epidemiological distribution characteristics of symptoms. This network analysis framework based on symptom severity can more accurately identify core driving and bridging symptoms and provide a theoretical basis for developing precise symptom management strategies.
**Comment 4:Gender and smoking network comparison: authors did not mention about the methods of how to conduct this, please be specific and provide very details of this information in the method section. Also, why gender and smoking are choosen? add background about this subgoup analysis in the INTRODUCTION. Also, add tables of this results including statistical value and p values**

**Comment 4.1:.Also, why gender and smoking are choosen? add background about this subgoup analysis in the INTRODUCTION.**

**Reply:** Thank you for your valuable comments. We conducted linear regression analyses with total symptom severity as the dependent variable and participant characteristics as the independent variable (see Table 1 for details). The results showed that gender and smoking history had a significant effect on total symptom severity (P < 0.001), and therefore gender and smoking history were analyzed as subgroups in the network analysis. In addition, we have included in the introduction the reasons for choosing between gender and smoking history. Please refer to lines 179-210 of the revised manuscript:

Table 1 Characteristics of the participants and linear regression analysis of overall symptom severity (n =249)

| Variables | Mean (SD) or n (%) | β | *P* |
| --- | --- | --- | --- |
| **Age(years)** | | | |
| ＜60* | 77（30.9%） |  |  |
| ≥60 | 172（69.1%） | -0.053 | 0.313 |
| **Gender** | | | |
| Male* | 187（75.1%） |  |  |
| Female | 62（24.9%） | 0.386 | ＜0.001 |
| **Body mass index(kg/m2)** | | | |
| waste away* | 62（24.9%） |  |  |
| normal | 145（58.2%） | 0.075 | 0.218 |
| overweight | 25（10.0%） | 0.065 | 0.286 |
| obese | 17（6.8%） | 0.041 | 0.461 |
| **Education level** | | | |
| Primary and below* | 159（63.9%） |  |  |
| Secondary schools | 50（20.1%） | 0.056 | 0.313 |
| High school and above | 40（16.1%） | -0.157 | 0.004 |
| **Smoking history** | | | |
| No* | 134（53.8%） |  |  |
| Yes | 115（46.2%） | -0.532 | ＜0.001 |
| **Residence** | | | |
| Urban* | 134（53.8%） |  |  |
| rural | 115（46.2%） | -0.035 | 0.504 |
| **Tumor stage** | | | |
| I* | 1（0.0%） |  |  |
| II | 10（0.4%） | -0.004 | 0.983 |
| III | 58（23.2%） | -0.267 | 0.441 |
| IV | 180（72.3%） | -0.229 | 0.527 |
| **Pathological type** | | | |
| small cell carcinoma* | 56（22.5%） |  |  |
| squamous carcinoma | 85（34.1%） | 0.117 | 0.084 |
| Adenocarcinoma | 101（40.6%） | 0.088 | 0.192 |
| large cell carcinoma | 7（2.8%） | 0.027 | 0.614 |
| **Family monthly income（yuan）** | | | |
| ≤3000* | 38（15.3%） |  |  |
| 3001-6000 | 57（22.9%） | 0.077 | 0.295 |
| 6001-9000 | 73（29.3%） | 0.077 | 0.316 |
| ≥9001 | 81（32.5%） | 0.074 | 0.338 |

Note:*is the reference group,R^2^=0.418,adjustedR^2^=0.373,F=9.181,*p*< 0.001.

The mechanism of heterogeneity of immune checkpoint inhibitor (ICI) treatment response in lung cancer and its difference in clinical manifestations is an important direction of current medical research. Among them, smoking status and gender differences, as key biological variables, modulate the interaction pattern between the immune microenvironment and the clinical symptom network through unique molecular mechanisms. From a pathophysiological perspective, smoking-induced bi-directional immunomodulation is of particular interest: tobacco combustion products (e.g., benzo(a)pyrene), through activation of the aromatic hydrocarbon receptor (AhR) signaling pathway, can promote the up-regulation of programmed death ligand 1 (PD-L1) expression and the recruitment of myeloid-derived suppressor cells (MDSCs) to form an immunosuppressive micro environment[36]. This paradoxical effect may lead to a unique symptom interaction characterized by smoking patients - both a treatment response advantage due to elevated TMB and a synergistic worsening of respiratory-consumptive symptom clusters (coughing, shortness of breath, weight loss) exacerbated by chronic inflammation and comorbidities (e.g., COPD). In the gender difference dimension, the sex hormone-immunity axis regulatory mechanisms showed significant biological specificity. Estrogens enhance the antitumor activity of CD8+ T cells by modulating their mitochondrial metabolic reprogramming[37], whereas androgens reduce tissue repair capacity by inhibiting M2 polarization of tumor-associated macrophages (TAMs). This immunomodulatory difference may directly contribute to the gender dimorphism in symptom characterization. patients are more likely to present with fatigue-mood symptom clusters (e.g., fatigue, sadness, sleep disturbances), whereas somatic symptoms dominate men. A recent meta-analysis further confirmed that women exhibit superior pathologic remission rates in ICI monotherapy or combination therapy(major pathological responses(mPR): OR=1.82, 95%CI 1.13-2.93, p=0.01; pathological complete responses(pCR): OR=1.65, 95%CI 0.97-2.75, p=0.08)[37].These biological differences ultimately map to the topology of the clinical symptom network: smokers exhibit strong respiratory-metabolic symptom associations due to airway inflammation and a predisposition to systemic malignancy, while women have closed-loop feedback for emotional-somatic symptoms due to neuroendocrine-immune interactions, and this heterogeneity of the symptom network requires that healthcare providers implement precise symptom management strategies.

**Comment 4.2: Gender and smoking network comparison: authors did not mention about the methods of how to conduct this, please be specific and provide very details of this information in the method section.**

**Reply**: Thank you for your valuable comments on the manuscript. Specific methods for intergroup comparison of symptom networks have been added and codes for intergroup comparison of symptom networks have been provided. (see Supplementary file 1: the R language code). Please refer to lines 306-331 of the revised manuscript:

In the sensitivity analysis, we used a multilevel confounder control strategy: firstly, the covariates with significance level of P<0.001 in the multiple regression analysis were included in the network model as confounders; then we conducted Network Comparison Test (NCT) based on the Network Comparison Test package in R (version 2.2.1) to assess the heterogeneity of the network topology among different subgroups through 10,000 permutation test. A Network Comparison Test (NCT) was conducted based on the Network Comparison Test package in R (version 2.2.1) to assess the heterogeneity of the network topology among different subgroups through the 10,000 permutation test. The NCT analytical framework consists of an invariance test with three dimensions: 1) Network Structure Invariance: It is assumed that the overall topology of the network (i.e., the connection pattern between nodes) is not significant among different subgroups and that the overall topology of the network is not significant. Network Structure Invariance: It is assumed that the overall topology of the network (i.e., the connection pattern between nodes) is not significantly different between different subgroups, reflecting the group universality of symptom interaction patterns; 2) Global Strength Invariance: It is assumed that the overall connection strength (i.e., the sum of the absolute values of the weights of all edges) of the network is not significantly different between different subgroups, reflecting the group consistency in the overall loading level of symptom clusters; 3)Edge Strength Invariance. Edge Strength Invariance): It is assumed that the connection strength between specific symptom pairs does not differ significantly across subgroups and is the key symptom interaction pathway for identifying group specificity. The above methodological framework follows the best practice guidelines proposed by van Borkulo et al.(2022)[44], and the statistical robustness of the results is ensured by two-tailed tests and false discovery rate correction. (Symptom Network Intergroup Comparison Codes see Supplementary Document 1:R-language code.)

**Comment 4.3: Also, add tables of this results including statistical value and p values**

**Reply**: Thank you very much for your valuable comments on the reporting details of the symptom network analysis. We searched the literature related to the reporting format of between-group comparisons in network analysis and found that scholars are more likely to use graphs to represent the results of between-group differences in network invariance and global invariance. Therefore, we also report the results in graphical format, please refer to Figures 7 and 9 for the results of intergroup difference comparisons of symptom networks in this study.Figure 7 shows the comparison of network attributes between females and males. As shown in the right panel of Figure 7, according to the network invariance test, the NCT results showed that there was no significant difference in the topology of the symptom networks of males and females (M=0.279, *P*=0.275); as shown in the left panel of Figure 7, the global connection strength did not reach the significance level (S=0.972, *P*=0.104); Figure 9 shows the comparison of network attributes between those with and without a history of smoking. As shown in the right panel of Figure 9, according to the network invariance test, the NCT results showed that there was no significant difference in the topology of the network between those with a history of smoking and those without a history of smoking symptoms (M=0.187, *P*=0.772); and, as shown in the left panel of Figure 9, the global connectivity strength did not reach the level of significance (S=0.376, *P*=0.249). For a detailed description, see lines 455-484 of the

**Comment 5: discussion: please add how to intepret DIFFERENTLY between bridge and central node symptoms. Central node symptoms are most prevalent and severe? ? or just prevalence? or just severe? which dimention of symptoms are used inthis study? Authors clearly should mention the DIFFERENT INTEPRETATION between bridge and central node symptoms.
again, in mentioned in the method section, unclear whether author used SEVERITY or Prevalence (frequency) of ths symptoms.**

**Comment 5.1: which dimention of symptoms are used inthis study? again, in mentioned in the method section, unclear whether author used SEVERITY or Prevalence (frequency) of ths symptoms.**

**Reply:** Thank you very much for your valuable comments. In this study, symptom severity was used and a network analysis framework was constructed based on symptom severity for the following reasons (please refer to lines 332-353 of the revised manuscript):

The value of network analysis as an important methodological tool for complex systems research is not only limited to describing single-dimensional features such as the incidence or severity of symptoms, but also in revealing the dynamic interaction mechanisms among symptoms through topological indicators (e.g., centrality, intensity, tightness, and mediativity)[45]. This advantage is particularly prominent in the study of immunotherapy-related symptoms: as immune-related adverse events (irAEs) often present a subclinical persistence, the traditional dichotomous variable (presence/absence) loses the information about the gradient of symptom intensity, whereas continuous severity scores capture the dynamic evolution of the symptom load with greater precision. Moreover, from the methodological point of view: 1) the correlation between severity score and patients' quality of life was significantly higher than that of the incidence index, suggesting that it has more prognostic predictive value[46]; 2) the biased correlation network, also known as Gaussian graph model, constructed by network analysis requires the input of continuous variables, while the incidence data need to use Logistic network model[28]; And the severity gradient can reflect both the intensity dimension and the time dimension (e.g., fluctuation frequency, duration) of the symptoms, providing a richer basis for decision-making for dynamic intervention. Therefore, the symptom network constructed in this study reflects the covariate pattern of symptom severity rather than the epidemiological distribution characteristics of symptoms. This network analysis framework based on symptom severity can more accurately identify core driving and bridging symptoms and provide a theoretical basis for developing precise symptom management strategies.

**Comment 5.2: discussion: please add how to intepret DIFFERENTLY between bridge and central node symptoms. Central node symptoms are most prevalent and severe? ? or just prevalence? or just severe?**

**Reply:** We sincerely thank the reviewers for their profound insights into the differences in the interpretation of symptom network nodes. In view of the distinction between core node symptoms and bridge symptoms, we have supplemented the above differentiated interpretation framework in the discussion section.(Please refer to lines 533-547,594-618 in the revised manuscript.):

In the context of symptom networks, Central symptoms are key pivotal nodes in the symptom network, identified by Strength Centrality metrics. Strength centrality is defined as the sum of the absolute values of the edge weights of a node and its directly connected nodes, and the higher the value of strength centrality, the stronger the direct influence of the symptom in the network, and its fluctuations can be rapidly propagated to the neighboring symptoms through the shortest path[49]. Central symptoms have a dual character: not only are they at the top of both the symptom prevalence and severity distributions, but as high-influence nodes in the network, changes in their state can trigger cascading responses in symptom clusters through directly connected pathways. In this study, sadness (intensity centrality rs=8.40 with clinical covariates vs. intensity centrality rs=7.43 without clinical covariates), as a core node symptom, not only had a high prevalence of 70.2%, but its fluctuation also showed a significant covariation with the neighboring symptoms (e.g., fatigue r=0.76, cough r=0.64, nausea r=0.61, and distress r=0.60). and this correlation was not affected by variables such as gender and smoking history.

Bridge symptoms are pivotal nodes connecting different symptom clusters or disease subgroups in a network topology, identified by the bridge strength centrality metric. Bridge strength centrality is the sum of the absolute values of the edge weights of a node connecting different modules, with higher values indicating a stronger cross-module propagation of the symptom[58]. High bridge-strength symptoms (e.g., sadness in the present study), although not necessarily the highest in severity, act as cross-module propagation mediators and may trigger cascading deterioration of symptom clusters. In the present study, sadness (bridge strength centrality rs=6.66 with clinical covariates vs. bridge strength centrality rs=5.69 without clinical covariates) was the bridge symptom with the highest bridge strength in the symptom network, and the sadness associations encompassed respiratory symptom clusters of coughing, nausea, and fatigue, gastrointestinal symptom clusters and neuroperceptual symptom clusters, with a strong link between them. This implies that sadness is not only common to multiple symptom clusters but is also able to connect different symptom clusters.In addition, the symptoms of cough and nausea were also found to have a high bridging effect. Studies[58] have shown that by cutting off bridge symptoms, patients can be stopped from spreading from one disease to another. This is because bridge nodes or edges connect different clusters and have a higher ability to spread information in the network. Therefore, in symptom management for patients at this stage of the disease, sadness can be used as a high-impact intervention target throughout immunotherapy for lung cancer, and symptoms such as cough and nausea can be used as a sub-focus of intervention to develop a precise intervention program to weaken the information dissemination capacity of these bridge symptoms in the network, thus improving the effectiveness of symptom management for patients.
**Comment 6: all the figures I am very confusing is this based on the severity? or prevalence?**

**Reply:** Thank you very much for your valuable comments. The manuscript uses the severity of the symptoms and constructs a network analysis based on the severity of the symptoms for the following reasons (please refer to lines 332-353 of the revised manuscript):

The value of network analysis as an important methodological tool for complex systems research is not only limited to describing single-dimensional features such as the incidence or severity of symptoms, but also in revealing the dynamic interaction mechanisms among symptoms through topological indicators (e.g., centrality, intensity, tightness, and mediativity)[45]. This advantage is particularly prominent in the study of immunotherapy-related symptoms: as immune-related adverse events (irAEs) often present a subclinical persistence, the traditional dichotomous variable (presence/absence) loses the information about the gradient of symptom intensity, whereas continuous severity scores capture the dynamic evolution of the symptom load with greater precision. Moreover, from the methodological point of view: 1) the correlation between severity score and patients' quality of life was significantly higher than that of the incidence index, suggesting that it has more prognostic predictive value[46]; 2) the biased correlation network, also known as Gaussian graph model, constructed by network analysis requires the input of continuous variables, while the incidence data need to use Logistic network model[28]; And the severity gradient can reflect both the intensity dimension and the time dimension (e.g., fluctuation frequency, duration) of the symptoms, providing a richer basis for decision-making for dynamic intervention. Therefore, the symptom network constructed in this study reflects the covariate pattern of symptom severity rather than the epidemiological distribution characteristics of symptoms. This network analysis framework based on symptom severity can more accurately identify core driving and bridging symptoms and provide a theoretical basis for developing precise symptom management strategies.

**Comment 7: Please undergo professional English proofreading service.**

**Reply:** Thank you very much for your careful review of the manuscript and your valuable comments.We have accepted professional English proofreading services to ensure the quality of manuscripts.

**Reviewer #2**

**Reviewer #2: Providing a brief explanation of "nodal intensity" and "bridge intensity".
The "Conclusion" section could benefit from a more explicit statement about the implications of findings for clinical practice.**

**Comment 1: Providing a brief explanation of "nodal intensity" and "bridge intensity".
Reply:** We sincerely thank the reviewers for their profound insights into the differences in the interpretation of symptom network nodes. In view of the distinction between core node symptoms and bridge symptoms, we have supplemented the above differentiated interpretation framework in the discussion section.(Please refer to lines 533-547,594-618 in the revised manuscript.):

In the context of symptom networks, Central symptoms are key pivotal nodes in the symptom network, identified by Strength Centrality metrics. Strength centrality is defined as the sum of the absolute values of the edge weights of a node and its directly connected nodes, and the higher the value of strength centrality, the stronger the direct influence of the symptom in the network, and its fluctuations can be rapidly propagated to the neighboring symptoms through the shortest path[49]. Central symptoms have a dual character: not only are they at the top of both the symptom prevalence and severity distributions, but as high-influence nodes in the network, changes in their state can trigger cascading responses in symptom clusters through directly connected pathways. In this study, sadness (intensity centrality rs=8.40 with clinical covariates vs. intensity centrality rs=7.43 without clinical covariates), as a core node symptom, not only had a high prevalence of 70.2%, but its fluctuation also showed a significant covariation with the neighboring symptoms (e.g., fatigue r=0.76, cough r=0.64, nausea r=0.61, and distress r=0.60). and this correlation was not affected by variables such as gender and smoking history.

Bridge symptoms are pivotal nodes connecting different symptom clusters or disease subgroups in a network topology, identified by the bridge strength centrality metric. Bridge strength centrality is the sum of the absolute values of the edge weights of a node connecting different modules, with higher values indicating a stronger cross-module propagation of the symptom[58]. High bridge-strength symptoms (e.g., sadness in the present study), although not necessarily the highest in severity, act as cross-module propagation mediators and may trigger cascading deterioration of symptom clusters. In the present study, sadness (bridge strength centrality rs=6.66 with clinical covariates vs. bridge strength centrality rs=5.69 without clinical covariates) was the bridge symptom with the highest bridge strength in the symptom network, and the sadness associations encompassed respiratory symptom clusters of coughing, nausea, and fatigue, gastrointestinal symptom clusters and neuroperceptual symptom clusters, with a strong link between them. This implies that sadness is not only common to multiple symptom clusters but is also able to connect different symptom clusters.In addition, the symptoms of cough and nausea were also found to have a high bridging effect. Studies[58] have shown that by cutting off bridge symptoms, patients can be stopped from spreading from one disease to another. This is because bridge nodes or edges connect different clusters and have a higher ability to spread information in the network. Therefore, in symptom management for patients at this stage of the disease, sadness can be used as a high-impact intervention target throughout immunotherapy for lung cancer, and symptoms such as cough and nausea can be used as a sub-focus of intervention to develop a precise intervention program to weaken the information dissemination capacity of these bridge symptoms in the network, thus improving the effectiveness of symptom management for patients.
**Comment 2: The "Conclusion" section could benefit from a more explicit statement about the implications of findings for clinical practice.**

**Reply:** Thank you very much for your valuable comments. We have modified the conclusion according to your comments.(Please refer to lines 646-661 in the revised manuscript.):

In this study, a network analysis Gaussian graph model was used to construct the symptom network of lung cancer immunotherapy survivors, and sadness was found to have a dual topological feature: It was both a core node symptom and a key bridge symptom, suggesting that it should be a priority target for multidimensional interventions. Analysis of symptom dimensions revealed that nausea had the highest incidence and that fatigue severity was the most significant. Although subgroup analyses did not reveal differences in network structure by gender and smoking history, males and smokers demonstrated higher network density. This study suggests that multimodal interventions targeting sadness may lead to a reduction in global network strength and simultaneous improvement in respiratory, gastrointestinal, and neuroperceptual symptom clusters. Healthcare professionals should pay attention to the significant impact of negative “sadness” emotions on mental health and physical symptoms in lung cancer immunotherapy survivors. In the future, time series network analysis and Mendelian randomization are needed to dynamically track symptom evolution and validate causal effects, providing evidence-based pathways for precision medicine.

References

4.	Rajapakse P: An Update on Survivorship Issues in Lung Cancer Patients. World J Oncol 2021, 12(2-3):45-49, DOI: 10.14740/wjon1368.

5.	Hirpara DH, Gupta V, Davis LE, Zhao H, Hallet J, Mahar AL, Sutradhar R, Doherty M, Louie AV, Kidane B *et al*: Severe symptoms persist for Up to one year after diagnosis of stage I-III lung cancer: An analysis of province-wide patient reported outcomes. Lung Cancer 2020, 142:80-89, DOI: 10.1016/j.lungcan.2020.02.014.

6.	Sung MR, Patel MV, Djalalov S, Le LW, Shepherd FA, Burkes RL, Feld R, Lin S, Tudor R, Leighl NB: Evolution of Symptom Burden of Advanced Lung Cancer Over a Decade. Clin Lung Cancer 2017, 18(3):274-280.e276, DOI: 10.1016/j.cllc.2016.12.010.

7.	Chuanmei W, Heling Z, Yanhua J, Yaqin W, Jia L, Fan Y, Yaqian F, Xing W, Qiang L, Wei D *et al*: Preoperative Self-Reported Symptom Burden and Quality of Life of Lung

Cancer Patients: A Cross-Sectional Study. J Cancer Control Treat 2021, 34(03):234-240, DOI: 10. 3969 /j. issn. 1674-0904. 2021. 03. 008.

8.	Jie L, Huiling Z, Lijun S, Xuejiao M, Xiaoyue W, Hongsheng L: Current status of research on symptom clusters in lung cancer patients at different treatment stages. Modernization of Traditional Chinese Medicine and Materia Materia-World Science and Technology 2020, 22(04):926-930, DOI: 10.11842/wst.20200629005.

9.	Yafen Y, Ting W, Feng H, Yuwei C, Mei D: Research progress on symptom burden and nursing countermeasures in elderly lung cancer patients. General practice nursing 2024, 22(15):2851-2855, DOI: 10.12104/j.issn.1674-4748.2024.15.019.

19.	Chen K, Yang D, Li F, Gao L, Tian Y, Xu B, Xu X, Xu Q, Cao J: Changes in the symptom clusters of elderly patients with lung cancer over the course of postoperative rehabilitation and their correlation with frailty and quality of life: A longitudinal study. Eur J Oncol Nurs 2023, 67:102388, DOI: 10.1016/j.ejon.2023.102388.

20.	Li JJ, Li JR, Wu JM, Song YX, Hu SH, Hong JF, Wang W: Change in symptom clusters perioperatively in patients with lung cancer. Eur J Oncol Nurs 2021, 55:102046, DOI: 10.1016/j.ejon.2021.102046.

21.	Lijun S, Jie L, Simeng R, Xuejiao M, Surui Y, Daorui L: A study of symptom burden and symptom clusters at the end of chest radiotherapy in 220 lung cancer patients. Modernization of Traditional Chinese Medicine and Materia Medica-World Science and Technology 2023, 25(06):2140-2146, DOI: 10.11842/wst.20220119007.

22.	Luo Y, Zhang L, Mao D, Yang Z, Zhu B, Miao J, Zhang L: Symptom clusters and impact on quality of life in lung cancer patients undergoing chemotherapy. Qual Life Res 2024, 33(12):3363-3375, DOI: 10.1007/s11136-024-03778-x.

23.	Ran W, Fei H: Longitudinal study on symptom groups of middle-aged and elderly lung cancer patients during chest

radiotherapy. Chinese Journal of Nursing 2023, 58(13):1596-1602, DOI: 10.3761/j.issn.0254-1769.2023.13.010.

24.	Chan CW, Richardson A, Richardson J: A study to assess the existence of the symptom cluster of breathlessness, fatigue and anxiety in patients with advanced lung cancer. Eur J Oncol Nurs 2005, 9(4):325-333, DOI: 10.1016/j.ejon.2005.02.003.

25.	Gift AG, Stommel M, Jablonski A, Given W: A cluster of symptoms over time in patients with lung cancer. Nurs Res 2003, 52(6):393-400, DOI: 10.1097/00006199-200311000-00007.

28.	Epskamp S, Borsboom D, Fried EI: Estimating psychological networks and their accuracy: A tutorial paper. Behav Res Methods 2018, 50(1):195-212, DOI: 10.3758/s13428-017-0862-1.

31.	Han S, Zhang Y, Yang X, Li K, Zhang L, Shao Y, Ma J, Hu Y, Zhu Z, Zhang Y *et al*: Exploring core mental health symptoms among persons living with HIV: A network analysis. Front Psychiatry 2023, 14:1081867, DOI: 10.3389/fpsyt.2023.1081867.

32.	Papachristou N, Barnaghi P, Cooper B, Kober KM, Maguire R, Paul SM, Hammer M, Wright F, Armes J, Furlong EP *et al*: Network Analysis of the Multidimensional Symptom Experience of Oncology. Sci Rep 2019, 9(1):2258, DOI: 10.1038/s41598-018-36973-1.

33.	Borsboom D, Rhemtulla M, Cramer AO, van der Maas HL, Scheffer M, Dolan CV: Kinds versus continua: a review of psychometric approaches to uncover the structure of psychiatric constructs. Psychol Med 2016, 46(8):1567-1579, DOI: 10.1017/s0033291715001944.

34.	Luo Y, Luo J, Su Q, Yang Z, Miao J, Zhang L: Exploring Central and Bridge Symptoms in Patients with Lung Cancer: A Network Analysis. Semin Oncol Nurs 2024, 40(3):151651, DOI: 10.1016/j.soncn.2024.151651.

35.	Xiaobo W, Huihong W, Lijia H, Xinyan W, Mingxi Z, Qing L, Hong J, Danfeng G: An investigative study of perioperative core symptoms of lung cancer based on network analysis. Journal of nurses training 2023, 38(24):2333-2339, DOI: 10.16821/j.cnki.hsjx.2023.24.022.

36.	Rizvi NA, Hellmann MD, Snyder A, Kvistborg P, Makarov V, Havel JJ, Lee W, Yuan J, Wong P, Ho TS *et al*: Cancer immunology. Mutational landscape determines sensitivity to PD-1 blockade in non-small cell lung cancer. Science 2015, 348(6230):124-128, DOI: 10.1126/science.aaa1348.

37.	Zhang X, Cheng L, Gao C, Chen J, Liao S, Zheng Y, Xu L, He J, Wang D, Fang Z *et al*: Androgen Signaling Contributes to Sex Differences in Cancer by Inhibiting NF-κB Activation in T Cells and Suppressing Antitumor Immunity. Cancer Res 2023, 83(6):906-921, DOI: 10.1158/0008-5472.Can-22-2405.

39.	Eigentler TK, Hassel JC, Berking C, Aberle J, Bachmann O, Grünwald V, Kähler KC, Loquai C, Reinmuth N, Steins M *et al*: Diagnosis, monitoring and management of immune-related adverse drug reactions of anti-PD-1 antibody therapy. Cancer Treat Rev 2016, 45:7-18, DOI: 10.1016/j.ctrv.2016.02.003.

40.	Shalhout SZ, Kaufman HL, Emerick KS, Miller DM: Immunotherapy for Nonmelanoma Skin Cancer: Facts and Hopes. Clin Cancer Res 2022, 28(11):2211-2220, DOI: 10.1158/1078-0432.Ccr-21-2971.

44.	van Borkulo CD, van Bork R, Boschloo L, Kossakowski JJ, Tio P, Schoevers RA, Borsboom D, Waldorp LJ: Comparing network structures on three aspects: A permutation test. Psychol Methods 2023, 28(6):1273-1285, DOI: 10.1037/met0000476.

45.	Zheng Z, Tiantian H, Yilin J, Jiamin H, Junwen Y, Zhongfang Y: Basic concepts of symptom networks and its implementation in symptom management. Journal of Continuing Nursing Education 2023, 38(24):2214-2218+2224, DOI: 10.16821/j.cnki.hsjx.2023.24.002.

46.	Miaskowski C, Barsevick A, Berger A, Casagrande R, Grady PA, Jacobsen P, Kutner J, Patrick D, Zimmerman L, Xiao C *et al*: Advancing Symptom Science Through Symptom Cluster Research: Expert Panel Proceedings and Recommendations. J Natl Cancer Inst 2017, 109(4), DOI: 10.1093/jnci/djw253.

49.	Hofmann SG, Curtiss J, McNally RJ: A Complex Network Perspective on Clinical Science. Perspect Psychol Sci 2016, 11(5):597-605, DOI: 10.1177/1745691616639283.

58.	Jones PJ, Ma R, McNally RJ: Bridge Centrality: A Network Approach to Understanding Comorbidity. Multivariate Behav Res 2021, 56(2):353-367, DOI: 10.1080/00273171.2019.1614898.
